# Treatment of Distal Phalanx Fracture Using Figure-of-Eight Suturing of the Nail

**DOI:** 10.7759/cureus.32438

**Published:** 2022-12-12

**Authors:** SK Mizanur, Bibin Selvin, Shivam Patel, Mukesh Phalak

**Affiliations:** 1 Orthopedics, Dr Dnyandeo Yashwantrao Patil (DY) Patil Medical College and Hospital, Pune, IND

**Keywords:** vertical figure-of-eight suture, crush injury, nail bed, distal phalanx fracture, nail bed injury

## Abstract

Nail bed avulsion injuries associated with a distal phalanx fracture are very common injuries occurring in industrial workers dealing with heavy machines. Often patients come with an open wound that needs a thorough wash and reduction of fracture with manipulation and traction along with suturing of the nail bed. The study was conducted on 20 patients with nail bed avulsion injuries associated with distal phalanx fracture. Patients were treated by a vertical figure-of-eight suturing of the nail plate with the nail bed after a reduction of the distal phalanx fracture. Patients were followed up periodically at a weekly interval for up to three months. This approach maintains excellent contact between the nail plate and matrix, limiting additional displacement of the nail plate. The vertical figure-of-eight suturing technique takes sutures and secures the nail by using the nail plate and the nail bed. It's simple, dependable, and easily taught to surgeons of any specialty. This procedure may be used even if the eponychium is not intact. It's a useful approach for anybody dealing with a nail bed avulsion injury that includes or excludes distal phalanx damage. This method of repair is straightforward, secure, and reproducible. It does not necessitate either the formal repair of damaged nail bed structures or the removal of the nail bed. This procedure can be performed in an outpatient setting with a local ring block, and the patient can return home the same day. Additionally, the patient can regain finger function within three months.

## Introduction

The germinal matrix is the progenitor of the nail plate, which is situated at the base of the nail and just beneath the nail plate where the sterile matrix lies. A small band of epithelium that extends between the posterior nail wall and the nail base is referred to as the eponychium. The hyponychium is an epithelial band stretched from the skin to the nail plate near the fingertip. The body of the nail is the broad structure between the lateral and medial nail folds [1]. Upper limb injury comprises one-third of all domestic injuries. Injuries to the fingertips are very common as the hands are the most exposed body part. The primary mechanism of injury is crush injury [2], which may present as a simple nail avulsion to compound phalanx fracture with nail bed injury [3]. Injuries were separated into those involving the nail alone and those with more extensive trauma to the soft tissue of the fingertip or with distal phalanx fracture. Only careful and standardized treatment can prevent nail dystrophies, for which assessment of the nail bed and germinal matrix and making a clinical decision for removal of the nail plate, repositioning the nail plate by suture repair, or application of a nail implant play crucial roles [4]. There is subconscious coordination between the nail bed and the fingertip during pinch and grasp, but there is conscious impairment in this coordination when the fingertip of the nail bed is injured, and the anatomy deviates from normal [5]. Fingertip injuries are one of the most common cases to the orthopedic surgeon since fingertips and digits of the hand are continuously exposed and frequently injured. Fingertip injuries involve varying degrees of fractures, ranging from the nail bed and nail plate to distal phalanx disruption [6]. The recommended treatment protocols are K wire fixation for the fracture and nail plate removal if the nail is avulsed [7]. In this study, fingertip traumas associated with fracture of the distal phalanx and nail bed disruption were managed with a vertical figure-of-eight suturing for the nail plate to nail bed after holding the fracture of the distal phalanx in anatomical position under the guidance of a C-arm to provide a dorsal splint for the injury to heal.

## Case presentation

**Figure 1:**
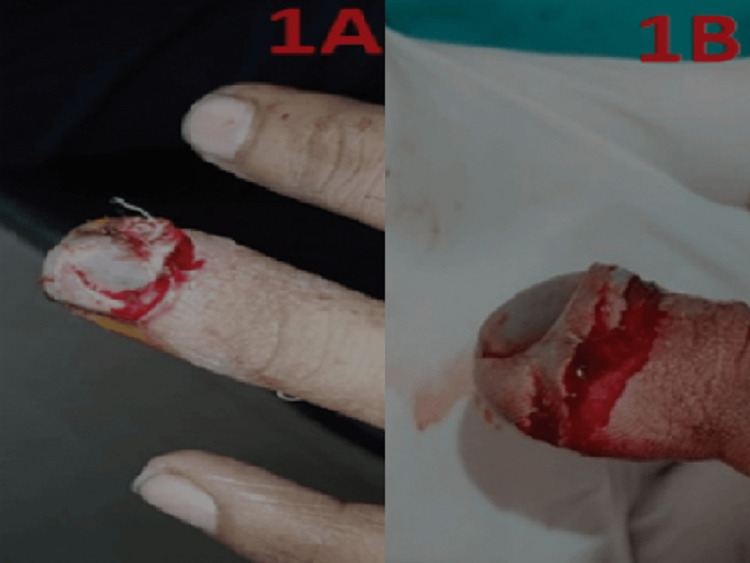
Nail bed avulsion injury (1A,1B)

The study was conducted between July 2020 and July 2021. Twenty patients (12 male and eight female) with fingertip injuries and distal phalanx fractures were sutured on the dorsal side using a vertical figure-of-eight suture with Ethilon (2-0) (Ethicon, Bridgewater, New Jersey), suturing the nail and the nail bed with fracture reduction of the distal phalanx but without fracture fixation. Each patient was managed by the same surgeon. Of the 20 patients, 15 had partial nail avulsion, whereas five had total nail avulsion from the nail bed in conjunction with a distal phalanx fracture (Figure 1).

The average age group was 34 (range 16-50) years. Table 1 shows the Pulp Nail Bone (PNB) classification of fingertip injuries.

**Table 1:** PNB classification of fingertip injuries in our study PNB - pulp nail bone, M - male, F - female

Age (years)	Sex	PNB classification
17	M	162
19	M	141
22	M	232
22	M	101
26	M	123
27	M	244
27	F	111
32	M	134
32	F	212
35	M	113
37	F	231
38	F	101
38	F	144
41	M	233
42	F	122
44	F	111
45	F	031
51	M	122
34	M	144
59	M	114

**Figure 2:**
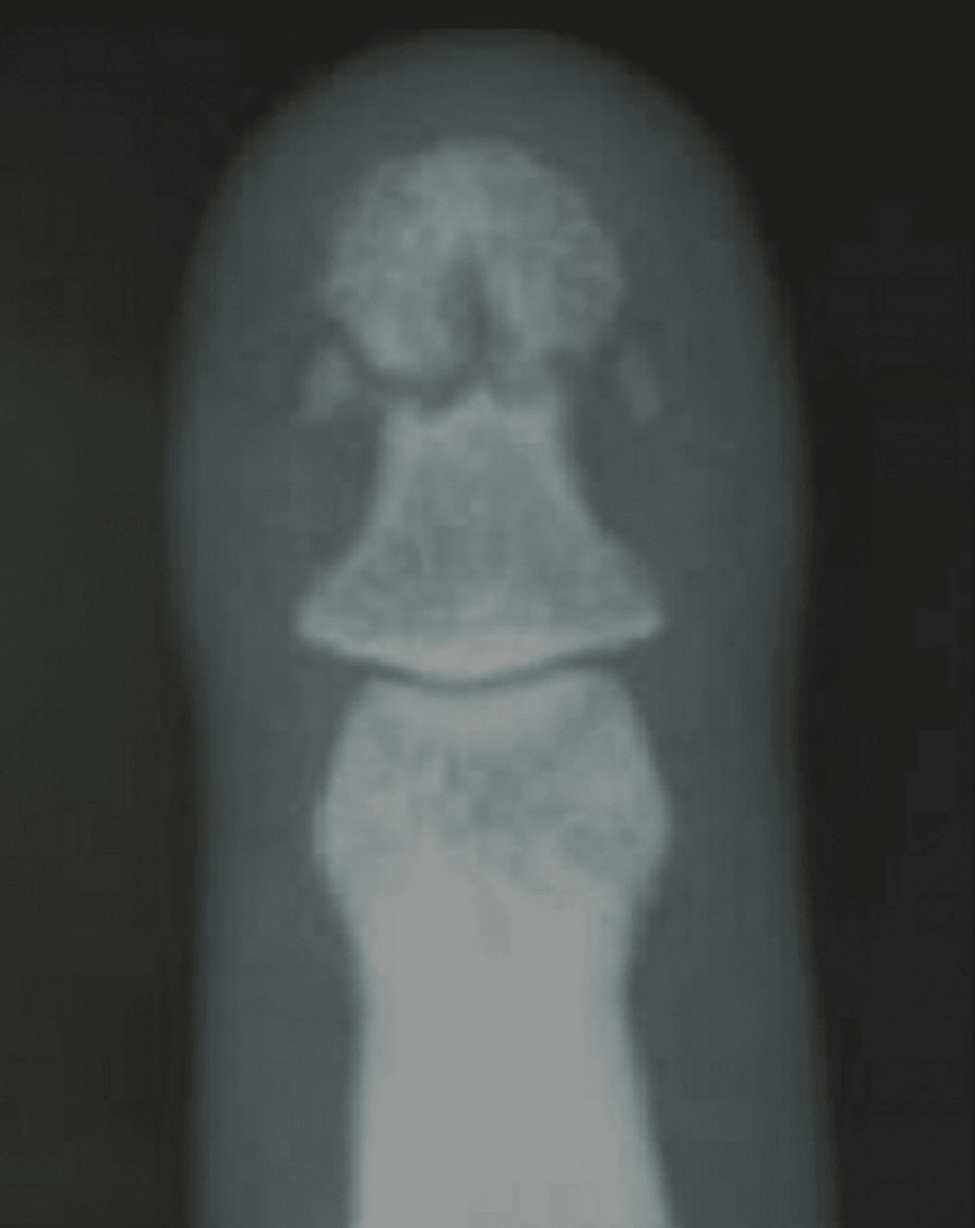
Pre-op X-ray of distal phalanx after reduction of the fracture

They were monitored weekly for the first three months. This study excluded cases of a full crush injury to the fingertip. After administering ring block anesthesia, a thorough wash of the damaged finger was performed, followed by sterile painting and draping. Any foreign object or hematoma was removed to properly expose the nail plate and its attachments. Traction and manipulation under C-arm supervision were used to reduce the distal phalanx fracture (Figure 2).

**Figure 3:**
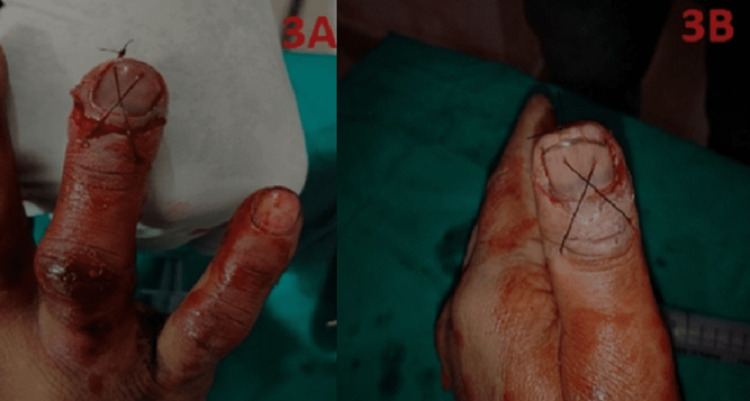
Vertical figure-of-eight suturing (3A, 3B)

Then, a figure-of-eight suture was preformed dorsally using nylon 3-0 sutures (Figure 3).

**Figure 4:**
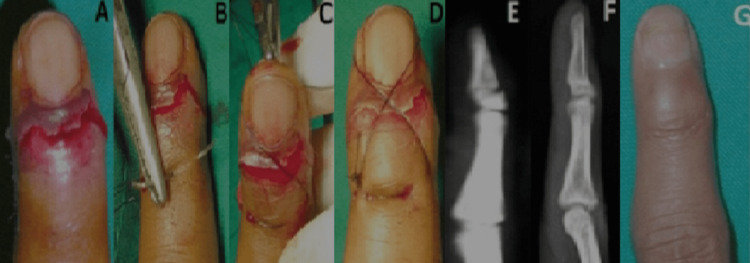
Case of a 21-year-old male with fingertip injury associated with nail bed avulsion (4A-4G) On examination, tenderness was present over the fingertip. On X-ray, a distal phalanx base fracture was observed. The patient was given a vertical figure-of-eight suture securing the nail plate with the nail bed after reducing the distal phalanx fracture. After four weeks, sutures were removed, and an X-ray was taken on follow-up. After six weeks, finger pinch movement was achieved.

In situations where the nail plate was avulsed concurrently with the fracture, the nail plate was repositioned anatomically and then sutured with 3-0 nylon sutures in a figure-of-eight pattern on the dorsum of the digit. To prevent injury to the nail bed during suturing, the skin was used as an anchor for proximal suturing. The suture is then spanned over the approximated nail plate and distally carried through the tip of the finger pulp to form a vertical eight loop, followed by a knot. The knot was secure enough to retain the nail plate's anatomical alignment with the nail bed. Precautions were taken to maintain the finger's distal vascular bundle. The vertical figure-of-eight suturing served as a dorsal splint, providing adequate support to maintain the reduction of the fingertip fracture fragment. Each case included a comment on the operation time (approximately 10 min). The surgical finger's vascular supply was monitored postoperatively. After confirming adequate perfusion, the dressing was performed with strict aseptic measures. Postoperative days two and five had their first and second dressings applied. Sutures were removed from the nail bed after four weeks or when the fingertip was sufficiently stable to maintain its normal anatomical orientation. At six weeks, finger mobilization with a pinch grasp was initiated. On average, the operation took approximately 20 (range 15-25) min. All patients underwent outpatient surgery. No patient required more than a half day of hospitalization. Follow-up was conducted on an outpatient department (OPD) basis for an average of three months. The patient's clinical result was contingent upon his/her functional outcome and capacity to squeeze without experiencing pain. The injured digit was compared with the normal finger using the visual analog scale. We concluded in this study that practically all patients recovered without major deformities. Furthermore, all fractures had a favorable functional outcome at the final follow-up. Each fingertip was stable, capable of pinching and grasping with the same strength as the opposing side and without deformation (Figure 4).

Two patients developed a postoperative infection, necessitating the amputation of the distal phalanx. The rest of the patients were able to resume ordinary work after an average of three months.

## Discussion

**Figure 5:**
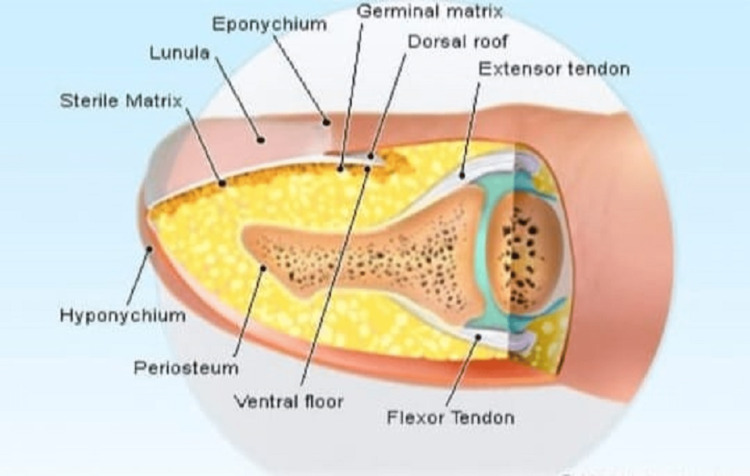
Nail bed anatomy

Fingertip injuries are the most common injuries associated with distal phalanx fractures and nail bed avulsion. The type of injury determines the location of the fracture; it may occur at the shaft, tuft, or base and may be associated with the total separation of the nail plate from the nail bed [8]. The primary goal of treatment is to anatomically relocate the nail plate over the nail bed, which will act as a dorsal splint for the distal phalanx fracture. Nail bed anatomy is shown in Figure 5.

Additionally, the sutured nail plate will aid in the formation of a smooth, compact nail. There is an extruding cell mass beneath the nail fold that assists the nail in growing distally, and the nail is created by compression of the fold. When the fold or bed of the nail is broken, it loses its usual function. Without dorsal support for the nail, the fingertip may never regain functional normality. Several therapeutic options for this type of injury have been attempted. The nail bed was delicately restored after removing any hematoma or foreign body. The relocated nail is supposed to aid in normal fingernail growth. Several techniques have been used to reposition the nail plate, the most common of which was anchoring the nail with the proximal fold or the distal hyponychium [9]. However, the already weakened soft tissue is subjected to additional trauma due to this operation. Suturing over the nail plate might be challenging at times, which is the reason some clinicians use acrylic adhesive for nail plate fixation. According to Foucher et al., postoperative nail fixation through the nail plate and nail bed should be maintained [10]. The smooth interface between the nail plate and the nail bed aids in the healing of the nail bed and also aids in the formation of the typical fingernail. It is recommended to leave the attached nail plate in place when suturing the nail bed. Historically, conventional nail bed repair has yielded mixed outcomes. According to a previous study, the transverse figure-of-eight suturing should be used to heal nail bed lesions [11]. This study aimed to determine the efficiency of the vertical figure-of-eight suturing of the nail bed as a dorsal splint for healing fingertip damage caused by distal phalanx fracture. There were 11 middle and proximal shaft fractures, nine distal phalanx tuft fractures, and various types of nail bed and nail plate avulsion in this study. After the reduction of the distal phalanx, we employed a vertical figure-of-eight suturing to secure the nail, which worked as a dorsal splint, providing sufficient support for the fractured area to heal while retaining normal anatomical alignment. The risk of developing a subungual hematoma is lowered by repairing the nail bed, resulting in decreased pain and tenderness over the fingertip. Additionally, suturing was used to reassemble the soft tissue surrounding the incision. The figure-of-eight suture was removed after four weeks or when the tip was stable.

## Conclusions

This repair method is straightforward, secure, and reproducible (nail regeneration). It does not require the formal repair of damaged nail bed structures or the removal of the nail bed. This procedure can be performed in an outpatient setting with a local ring block, and the patient can return home on the same day. Additionally, the patient can regain the functions of their fingers within three months.
